# Intermediate-Layer-Free Solid-Contact Ion-Selective Electrodes on Gold Microelectrode Arrays: A New Approach for Stable and Reproducible Potentials

**DOI:** 10.3390/ma19061238

**Published:** 2026-03-20

**Authors:** Klaudia Morawska, Karolina Pietrzak, Cecylia Wardak

**Affiliations:** 1 Department of Analytical Chemistry, Institute of Chemical Sciences, Faculty of Chemistry, Maria Curie-Sklodowska University, Maria Curie-Sklodowska Sq. 3, 20-031 Lublin, Poland; klaudia.morawska@mail.umcs.pl; 2 Centre for Advanced Laser Techniques, Institute of Physics, Bijenička cesta 46, 10000 Zagreb, Croatia; kpietrzak@ifs.hr

**Keywords:** ion-selective electrodes, gold microelectrode array, solid contact, potentiometry, potassium, nitrates

## Abstract

A new universal construction of intermediate-layer-free solid-contact ion-selective electrodes using a novel inner electrode, namely microelectrodes array composed of a large number of individual microelectrodes, was developed. This approach eliminates the need for a conventional solid-contact intermediate layer while maintaining excellent electrochemical performance. The studies were performed on two membrane model systems: potassium-ion-sensitive membranes based on valinomycin and nitrate-ion-sensitive membranes based on tridodecyldimethylammonium nitrate. In both cases, the membrane was applied directly onto the surface of the electrode substrate. The obtained results with such an ion-selective electrode based on a gold microelectrode array (GMA), a glassy carbon electrode (GCE), and a gold electrode (GE) were compared. It has been proven that, despite the lack of solid contact, whether in the form of an intermediate layer or as an addition directly to the membrane, ion-selective electrodes based on gold microelectrode arrays were characterized by very good analytical parameters. For those electrodes, a notable improvement in stability, reversibility, and repeatability of the electrode potential was observed and compared with electrodes using a glassy carbon disc electrode or a gold disc electrode as the electrode substrate. Thanks to the use of such an innovative electrode substrate, the final sensor preparation is shortened and simplified while maintaining good performance and stable readings.

## 1. Introduction

Nowadays, due to intensive socio-economic development, there is an increasing interest in protecting the environment and human health. At the same time, the requirements for testing methods to assess environmental conditions, human health, and product quality are becoming increasingly stringent. The search continues for methods that are inexpensive yet accurate and reliable, and that can provide results in a short period of time.

These conditions are fulfilled by potentiometry using ion-selective electrodes (ISEs), which additionally allow measurement in a continuous monitoring mode and directly in the test medium. Ion-selective electrodes are the largest and most widely used group of electrochemical sensors. Since the end of the last century, significant redevelopment of these sensors has been underway, driven by the introduction of solid-contact constructions—electrodes devoid of internal electrolyte solutions [1,2]. Solid-contact ion-selective electrodes (SC-ISEs), with their excellent detection performance, low cost, low energy consumption, simple equipment, and single-output parameters, have become the most promising biological and chemical sensing devices. In addition, SC-ISEs offer the advantages of easy miniaturization, even chip integration, easy carrying, strong stability, and more favourable ion detection in complex samples. They have been widely used in conjunction with portable, wearable, and intelligent detection devices, as well as in on-site analysis and timely monitoring in the fields of environment, industry, and medicine [3,4,5].

The most important element of ion-selective electrode construction is an ion-selective membrane (usually consisting of an ionophore, a plasticizer, and polyvinyl chloride as a polymeric matrix [6]), which, in conventional ion-selective electrodes, separates the sample solution from the internal solution [7]. In pursuit of sensors’ miniaturization and greater flexibility in constructing their shapes, an ion-selective membrane was placed directly on the material of the solid electrode, eliminating the internal solution. Its presence was associated with many limitations, including the need to store the electrodes vertically and the possibility of air entering the electrode. However, the combination of two materials with different conductivities resulted in an interface blockage. Placing a material with special properties (both ionic and electron conductivity) acting as a solid contact between the electrode surface and the ion-selective membrane layer made it possible to obtain sensors with satisfactory stability and reversibility of the electrode potential. In addition, it resulted in a significant improvement in the mechanical resistance of ISEs, making their transport and storage easier [8].

The performance of the solid contact ion-selective electrodes, in which the internal solution has been eliminated, depends not only on the properties of the ion-sensitive membrane, but also on the type of the internal electrode and the intermediate layer placed between this electrode and the membrane (solid contact) [9,10]. So far, the main focus has been on the production and application of new active substances (ionophores) responsible for the membrane selectivity for selected ions or the development of new variations in solid contacts aimed at achieving devices with improved analytical parameters, while simultaneously being more cost-effective and user- and eco-friendly. Many works prove that the use of solid contact material allows a significant improvement in electrode parameters, especially the stability of indications in the short and long term [11,12,13]. However, the solution is not without drawbacks.

Electrodes with solid-contact conductive polymers are often sensitive to changes in pH, light, or the presence of gases, such as oxygen or carbon dioxide [14]. Moreover, an aqueous film will form between the membrane and the inner contact. The composition of this layer changes during the use of the electrode, which causes instability of its readings [15].

The second large group of materials used as solid contact are carbon-based nanomaterials. In this case, there is a problem with particle aggregation and the need to use additional dispersants [16,17]. Moreover, electrodes based on carbon nanomaterials may be sensitive to changes in the redox potential of the sample solution [18]. In each case, the solid contact material partially penetrates the structure of the ion-selective membrane, changing its properties, which may adversely affect sensor parameters [19].

An important structural element of SCISEs is the electrode substrate on which an ion-selective membrane is applied. There is a research gap in this area. There is little research on the influence of the substrate electrode on the parameters of the solid contact ion-selective electrode. Internal electrodes made of various materials, such as GCE [20], silver [21], copper [22], nickel [23], platinum [24], gold [25] or printed electrodes (SPCE) [26], are widely used in SCISEs for ion determination. To work properly, they require modification by applying a mediation layer, such as a conductive polymer, carbon nanomaterials, metal and metal oxide nanoparticles, which improves their electrochemical properties. The glassy carbon electrodes (GCEs) are used for the detection of ${\mathrm{Cu}}^{2+}$ [27], ${\mathrm{Ca}}^{2+}$ [28], ${\mathrm{Pb}}^{2+}$ [29], $K^{+}$ [30], ${\mathrm{Cl}}^{-}$ [31], and ${\mathrm{NO}}_{3}^{-}$ [32], among others, but their effectiveness depends on a suitable mediation layer. Silver electrodes are used in both liquid- and solid-contact electrodes, such as for $K^{+}$ determination [33,34]. New designs include Ag wires coated with Ag nanoparticles, used in SCISEs sensitive to selenates [35] and arsenates [36]. Cu [37], Ni [38], Pt [39], and Au [40] wires are used as substrates, but require coating with conductive layers, i.e., MWCNTs or materials applied by electrodeposition or drop-casting [41]. Graphene-based ISEs include various forms of graphene, such as three-dimensional graphene sponge [42], graphene sheets [43], and laser-induced graphene used in NO_3_^−^-ISEs [44] and in a multifunctional $K^{+}$ detection platform in sweat [45]. Printed electrodes and those made of unusual materials (e.g., cotton threads or paper) also require additional coating, such as conductive ink, as shown in studies of ${\mathrm{Na}}^{+}$, $K^{+}$, ${\mathrm{Ca}}^{2+}$, and ${\mathrm{Cl}}^{-}$ sensitive electrodes [46], as well as in graphene paper-based ISEs for ${\mathrm{Pb}}^{2+}$ determination [47]. Despite the wide range of available substrates, new variants that offer increasingly better analytical properties are constantly being developed. However, the SCISEs proposed so far require additional modifications so in order to avoid additional costs and time for electrode preparation, work is being done on electrodes that will not require additional modification steps (besides applying an ion-sensitive membrane).

In this work, we present research on developing a new universal design for solid-contact ion-selective electrodes using a new type of internal electrode, namely a microelectrode array composed of numerous individual microelectrodes. It is well known that problems with the potential instability of coated wire or coated disc ion-selective electrodes are related to the inefficient charge transport process at the membrane/electrode substrate interface [48]. Therefore, using microelectrodes instead of macroelectrodes seems to be a good solution for ion-selective electrodes. It has been experimentally confirmed that charge transport at the interface of the microelectrode/water solution is much more effective than in a macroelectrode and allows analysis in highly resistive solutions and even without the addition of a basic electrolyte [49,50]. The use of a single microelectrode as an internal conducting electrode is possible, but the resulting ion-selective electrode has very high resistance, which causes many measurement difficulties. The use of a microelectrode array as an electrode substrate allows the advantages of the microelectrode to be exploited while avoiding its drawbacks, i.e., high resistance causing unstable readings.

Using a new type of substrate in ion-selective electrodes will ensure an efficient charge transfer process between the membrane and the internal electrode, effectively eliminating the main problem of SC-ISEs without using additional conductive materials. The absence of a mediation layer will result in better membrane adhesion to the substrate and extend the sensor’s lifespan. The absence of a mediation layer will result in better membrane adhesion to the substrate and extend the sensor’s lifespan. An additional benefit is that it simplifies and shortens the sensor preparation time and reduces its manufacturing costs. To the best of our knowledge, this is the first use of this type of substrate in the construction of ion-selective electrodes without an internal electrolyte.

## 2. Experimental Section

### 2.1. Experimental Apparatus and Measurements Setup

Potentiometric measurements were carried out for a cell consisting of a silver/silver chloride reference electrode with a double-junction system (Metrohm 6.0750.100) and tested ion-selective electrodes differing in the type of internal electrode. A 16-channel data acquisition system (Lawson Labs, Inc., Malvern, PA, USA) connected to a computer with appropriate software was used to obtain and collect electrode potential value data. The electromotive force measurements were obtained in the mixed solutions with a magnetic stirrer at room temperature. Chronopotentiometric measurements were carried out in a system consisting of three electrodes: our tested ISEs as the working electrode, a silver/silver chloride reference electrode with KCl (3 mol L^−1^) as a reference electrolyte (Metrohm 6.0733.100) and a glassy carbon (GC) rod 2 mm/65 mm (Metrohm, Herisau, Switzerland) as an auxiliary electrode. A solution of KNO_3_ with a concentration of 10^−1^ mol L^−1^ was used. An AUTOLAB electrochemical analyzer (Eco Chemie, Utrecht, The Netherlands) controlled by NOVA 2.1 software was used as the measuring device. The chronopotentiometric graphs were obtained by applying a constant current of +100 nA for 60 s, followed by −100 nA for another 60 s. The real image of the gold microelectrodes array surface was taken using a NIKON ECLIPSE MA200 optical microscope (Nicon, Tokyo, Japan). The image was taken using the reflected-light method with an increased depth of field.

### 2.2. Materials and Reagents

The materials necessary to obtain the membrane mixtures were as follows: valinomycin (potassium ionophore I) (Aldrich, St. Louis, MO, USA), potassium tetrakis(p-chlorophenyl) borate (KT_P_ClPB) (Fluka, Buchs, Switzerland), low molecular weight poly(vinyl) chloride (PVC) (Aldrich, Steinheim, Germany), bis(2-ethylhexyl) sebacate (DOS) (Fluka, St. Louis, MO, USA) for potassium-sensitive electrodes and tridodecyldimethylammonium nitrate (TDMANO_3_) (Aldrich, St. Louis, MO, USA) and o-nitrophenyl octyl ether (NPOE) (Aldrich, St. Louis, MO, USA) for nitrate-sensitive electrodes. Tetrahydrofuran (THF) obtained from Chempur (Piekary Śląskie, Poland) was used as the organic solvent necessary for the membrane mixture’s preparation.

Three types of electrode substrate were used: a new gold microelectrode array (GMA), a glassy carbon disc (3 mm diameter) electrode (GCE), and a gold disc (3 mm diameter) electrode (GE).

**Figure 1:**
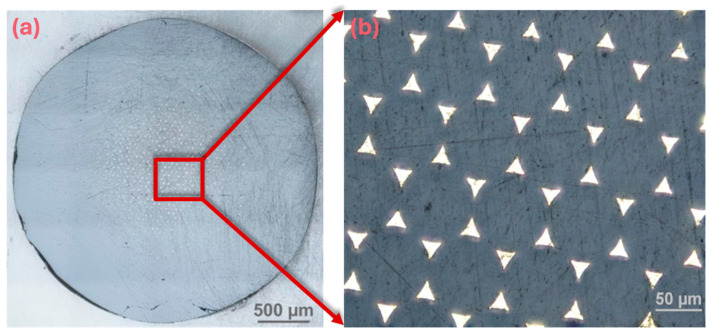
Real image of the gold microelectrodes array surface obtained using an optical microscope, whole substrate (**a**) and magnified fragment (**b**).

**Figure 2:**
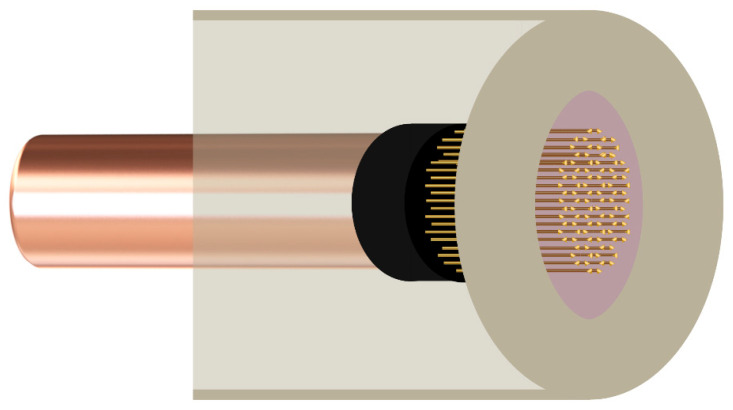
Cross-section diagram of a gold microelectrode array.

The GMA, which was used for the first time as an internal electrode in potentiometry, is composed of a large number of individual microelectrodes (about 400 in this case) with a suitable ordered structure and geometry that can be obtained relatively easily by using silica preforms with holes of defined shape, number and distribution. The preparation of such electrode substrates is relatively simple. It consists of filling these preforms with molten gold of suitable purity using sufficiently high pressure. A similar procedure is described in the literature [51,52]. An example of such an electrode substrate is shown in Figure 1, and the graphic presentation of GMA in cross-section is presented in Figure 2.

The main ion salt (KNO_3_) and the salts necessary for the selectivity tests of sensors: LiNO_3_, NaNO_3_, Mg(NO_3_)_2_, Ca(NO_3_)_2_ and K_2_SO_4_, K_2_CO_3_, CH_3_COOK and KF were obtained from Fluka. Distilled and deionized water was used to prepare the salt solutions.

### 2.3. Fabrication of the Ion-Sensitive Membrane

Two types of membrane mixtures were prepared with the following compositions. For the electrodes sensitive to $K^{+}$, the membrane cocktail contained: 3% valinomycin, 1% KTPClPB, 32% PVC and 64% DOS, while for ${\mathrm{NO}}_{3}^{-}$: 6% TDMANO_3_, 32% PVC and 62% NPOE. After the components were weighed, THF (1 mL per 0.1 g of membrane components) was added, and the mixtures were placed in an ultrasonic bath for 30 min to thoroughly mix. The membrane mixtures prepared this way were ready to be spotted on the specially prepared electrode surfaces.

### 2.4. Design and Preparation of Solid Contact Ion-Selective Electrodes

The preparation of the internal electrodes consisted of appropriate mechanical polishing and cleaning of their surfaces. The surface of the GCE was polished with an alumina powder (size 0.3 µm) wetted with distilled water. On the other hand, a moist fine-grit sandpaper (grain 5000) was used to polish the surfaces of the GE and the GMA. Then, the electrodes were rinsed with distilled water and placed in an ultrasonic bath to remove residual impurities and abrasive material. The electrodes were rinsed again with distilled water and degreased by immersion in THF. Subsequently, the solvent was allowed to evaporate. The membrane mixture was spotted onto the dry electrode surface prepared in such a way and placed in a vertical position in the stand. Three layers of the membrane mixture were dropped, 25 µL each time, with an interval of half an hour. The finished electrodes were allowed to dry overnight, and the next day they were immersed in a 10^−3^ mol L^−1^ KNO_3_. Throughout their use, the electrodes were kept in the same solution and stored in a dark place to avoid any potentially undesirable effects of light on the properties of the polymer membrane.

## 3. Results and Discussion

Extensive studies have been carried out to compare the analytical parameters of electrodes differing only in substrate material: gold microelectrode array (GMA), glassy carbon electrode (GCE), and gold disc electrode (GE). Electrodes with a spotted ion-selective membrane sensitive to $K^{+}$ (GMA/ISM-$K^{+}$, GCE/ISM-$K^{+}$ and GE/ISM-$K^{+}$) and ${\mathrm{NO}}_{3}^{-}$ (GMA/ISM-${\mathrm{NO}}_{3}^{-}$, GCE/ISM-${\mathrm{NO}}_{3}^{-}$ and GE/ISM-${\mathrm{NO}}_{3}^{-}$) ions were constructed. No intermediate layer was used between the electrode material and the membrane layer. In order to compare the properties of the obtained sensors, measurements were made, including obtaining electrode calibration curves, from which information on slope, linearity, and detection limits for the electrodes sensitive to the respective ions was obtained. In addition, the stability of the electrode potential and selectivity were also investigated. Further tests of the electrodes based on the GMA substrate were carried out for the water-layer test; measurements were performed by chronopotentiometry, and the analytical usefulness of the electrodes was checked in the analysis of real samples.

### 3.1. Potentiometric Characteristics of the Tested Electrodes

**Figure 3:**
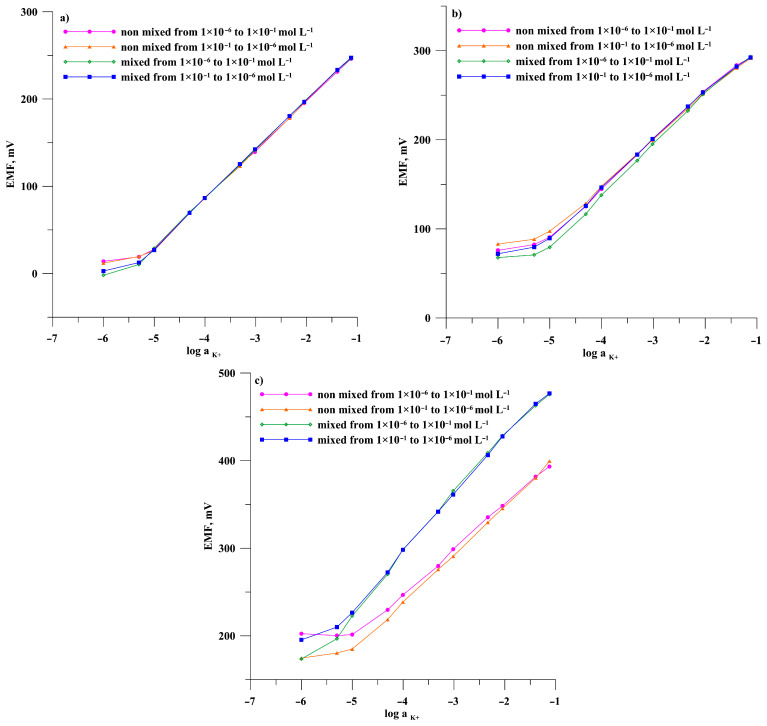
Calibration curves for electrodes: (**a**) GMA/ISM-K^+^, (**b**) GCE/ISM-K^+^, and (**c**) GE/ISM-K^+^ determined in the direction of increasing and then decreasing concentrations of the main ion for unmixed and mixed solutions, respectively.

**Figure 4:**
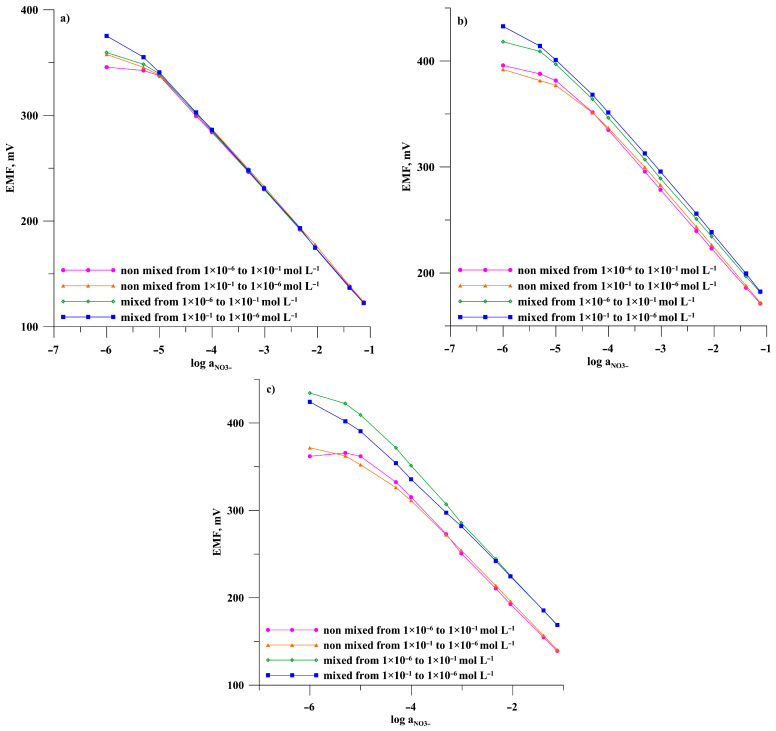
Calibration curves for electrodes: (**a**) GMA/ISM-${\mathrm{NO}}_{3}^{-}$, (**b**) GCE/ISM-${\mathrm{NO}}_{3}^{-}$, (**c**) GE/ISM-${\mathrm{NO}}_{3}^{-}$ in the direction of increasing and then decreasing concentrations of the main ion for unmixed and mixed solutions, respectively.

Electromotive force (EMF) measurements were carried out for a cell consisting of a reference electrode and tested electrodes in a main ion solution (KNO_3_) in the concentration range of 10^−6^–10^−1^ mol L^−1^. Calibrations were performed in two modes –in the direction of increasing concentrations and the direction of decreasing concentrations, each mode performed in mixed and unmixed solutions, respectively. The purpose of this procedure is to check the conditions under which the measurement is carried out for the repeatability of electrode readings. The obtained results are presented in Figure 3 and Figure 4 for potassium and nitrate electrodes, respectively.

The calibration curves for the individual calibration modes were used to determine the electrode parameters, i.e., the linearity range, the slope of the characteristic, the detection limit, and the potential E^0^. The data obtained are summarized in Table 1 and Table 2 for the potassium and nitrate electrodes, respectively.

By analyzing the course of the calibration curves of potassium electrodes shown in Figure 3a–c and nitrate electrodes shown in Figure 4a–c, taken with and without mixing solutions at lower and higher concentrations, it can be concluded that the type of substrate electrode significantly affects the repeatability of the electrode readings. The most reproducible calibration curves were observed for the GMA/ISM-K^+^ and GMA/ISM-NO_3_^−^ electrodes in which the ion-selective membrane was deposited on a gold microelectrode array. Consequently, the characteristics slope and potential E^0^ for these electrodes (Table 1 and Table 2) also have very similar values regardless of the calibration mode. The extreme slope values differ from 0.54 mV/decade for the potassium electrode and 0.66 mV/decade for the nitrate electrode. The maximum differences in E^0^ potential values were 1.47 and 2.4 mV for the electrodes GMA/ISM-K^+^ and GMA/ISM-NO_3_^−^, respectively. Changes in the position of individual calibration points are observed only for solutions with concentrations below 1 × 10^−5^ mol L^−1^. For electrodes made with disc macroelectrodes, the reliability of the indications is significantly worse, but to different extents for GCE and GE-based ISEs. Significant differences in calibration curves were observed between mixed and unmixed solutions for electrodes prepared using a gold disc substrate. The slope values differed by as much as 15.95 mV/decade for the GE/ISM-K^+^ and 6.66 for the GE/ISM-NO_3_^−^. Similarly, large changes were observed in E^0^ potential values determined for the different calibrations of these electrodes. Better repeatability of readings and less scatter in the characteristics slope and potential E^0^ values were observed for electrodes based on the GCE substrate. Moreover, the observed differences are much greater than those observed with electrodes based on the GMA substrate. The mixing of the solutions affects the linearity ranges of the calibration curves. For each electrode, regardless of the substrate used, the linear range is approximately half an order of magnitude wider at lower concentrations in mixed solutions.

Since the only difference in the design of the SCISES under investigation is the type of internal electrode, the observed differences in the calibration curves depending on the measurement conditions—i.e., with or without stirring—are most likely due to the geometry and properties of the electrode substrate. The macroelectrode operates under conditions of linear diffusion. In this case, under static conditions (without mixing), a thicker diffusion layer forms, which may restrict charge flow, whilst during mixing, charge transport occurs more efficiently and the thickness of the diffusion layer is reduced by the continuous movement of the solution. In an electrode consisting of a gold microelectrode array (GME), charge transport results from radial diffusion; convection does not have such a significant effect on the electrode response, as it is dispersed across the subunits of the microelectrode array. Consequently, the presence or absence of mixing does not significantly affect the signal.

### 3.2. Verification of the Potential Stability of the GCE and GEs in Relation to the New Type of GMA Electrode Without an Intermediate Layer

**Figure 5:**
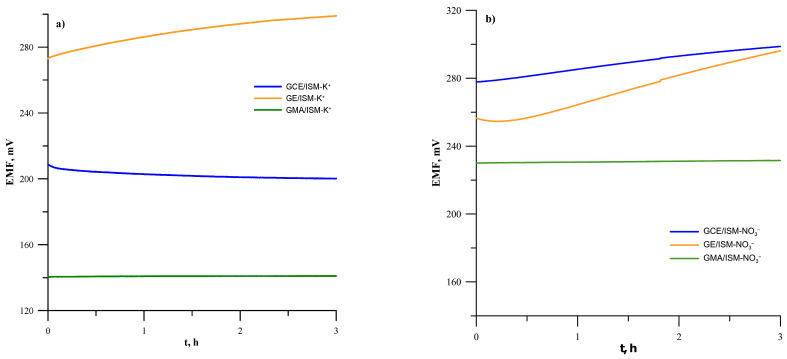
Stability of the potential of the tested electrodes measured during 3 h in 1 × 10^−3^ mol L^−1^ KNO_3_ for potassium (**a**) and nitrate (**b**) electrodes.

The potential stability of ion-selective electrodes is a very important parameter that greatly affects the accuracy of determination results. Potential stability problems often occur with coated disc or coated wire electrodes, for which additive conductive materials are used to achieve sufficient stability of the potential during measurement [8]. For this reason, we conducted additional potential stability measurements on the tested electrodes. The short-term stability of the potential was investigated for 3 h in a KNO_3_ mixed solution with a concentration of 10^−3^ mol L^−1^. The obtained data is presented in Figure 5, where it can be seen that both electrodes on a GMA substrate show very good potential stability either during the first 10 min of measurement or during the rest of the experiment. The potential drift values calculated as the difference in potential (ΔE) per unit time (Δt) (Table 3) were very small and amounted to 0.0226 mV/min and 0.0151 mV during the first 10 min and 0.0024 and 0.0083 during the rest of the time for electrode GMA/ISM-K^+^ and GMA/ISM-NO_3_^−^, respectively. This is certainly an effect of the properties of the substrate electrode, as well as the effective charge transport between the membrane and a large number of microelectrodes. Such potential drift values are typical for solid-contact electrodes containing additional solid-contact material, e.g., a conductive polymer or a carbon nanomaterial such as carbon nanotubes. The other electrodes obtained on the basis of classical disc electrodes show a continuous potential drift in time, the value of which is significantly higher than that determined for GMA/ISM-K^+^ and GMA/ISM-NO_3_^−^ (Table 3). The improved signal stability observed for GME-based SCISEs compared to SCISEs based on Au and GCE macroelectrodes stems from differences in electrode geometry. Microelectrodes operate via radial diffusion, ensuring efficient charge transfer between the membrane and the substrate. The electrode reaches equilibrium more quickly, and as a result, its response is stable and less sensitive to local changes. In contrast, the macroelectrode operates on the basis of linear diffusion, making the signal more susceptible to changes in the thickness of the diffusion layer, which in turn leads to fluctuations and reduced stability.

### 3.3. Selectivity Studies

A significant parameter characterizing ion-selective electrodes is their selectivity, i.e., the possibility of determining the main ion in the presence of other ions in solution. Another part of the study was to determine whether the type of electrode substrate influences the selectivity of the electrode. The selectivity coefficients for the tested electrodes toward selected ions were estimated using the method of separable solutions based on the procedure given by Bakker in 2000 [53]. The electromotive force of the cell was measured first in solutions of the main ion with increasing concentrations to obtain a calibration curve, and then in aqueous solutions of various selected cations (Li^+^, Na^+^, Mg^2+^ and Ca^2+^ for electrodes with a membrane sensitive to K^+^ ions) and anions (SO_4_^2−^, CO_3_^2−^, CH_3_COO^−^ and F^−^ for electrodes with a membrane sensitive to NO_3_^−^ ions). The obtained functions were then extrapolated (a_i_ = a_j_ = 1 mol L^−1^) in order to calculate the value of $logK_{i,j}^{pot}$ using the formula:

$$logK_{i,j}^{pot}=\frac{E_{j}^{0}-E_{i}^{0}}{S}$$

where $E_{j}^{0}$, $E_{i}^{0}$—standard potential of the electrode in the solution of the interfering and main ion, respectively, S—the slope of the calibration curve in the main ion solution. The obtained $logK_{i,j}^{pot}$ values for all types of electrodes are presented in Table 4, where it can be seen that electrode selectivity did not depend on electrode substrate. This is understandable and fully consistent with the expected results, as the electrodes tested for a given ion used the same membrane with the same ionophore, whose properties have the greatest influence on the selectivity coefficient value. Given the selectivity coefficients, it can be seen that potassium electrodes exhibit very good selectivity. Of the ions studied, the relatively greatest interference is expected to come from ammonium ions. This fact must be taken into account when analyzing samples containing this ion in concentrations higher than those of potassium ions. In the case of nitrate electrodes, the strongest interfering impact was observed for ClO_4_^−^ ions. This is a well-known fact and a characteristic feature of highly lipophilic ions, which exhibit a high affinity for polymer membranes based on quaternary ammonium salts. In samples containing perchlorates, it is not possible to use nitrate electrodes without first removing the perchlorates. Nitrate analysis is usually performed on samples with very low nitrate content, as this does not cause interference.

### 3.4. The Effect of the Absence of an Intermediate Layer on the Results of the Water Layer Test in Electrodes with Different Internal Electrodes

**Figure 6:**
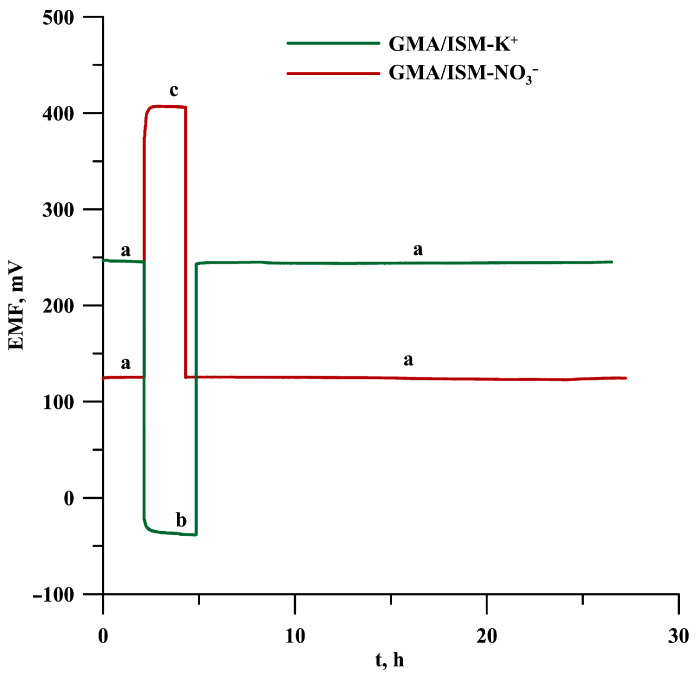
Water layer test performed for GMA/ISM-K^+^ and GMA/ISM-NO_3_^−^ electrodes: (a) 0.1 mol L^−1^ KNO_3_; (b) 0.1 mol L^−1^ NaNO_3_; and (c) 0.1 mol L^−1^ CH_3_COONa solution.

It has been shown that a thin aqueous layer can form between the internal electrode and the ion-sensitive membrane, which negatively affects measurements made with ISEs. The presence of an aqueous layer (the composition of which can change due to diffusion of components, etc.) not only adversely affects the stability of the potential but also its reversibility and reproducibility [15]. To determine whether such a form of interference in the form of an aqueous layer formed at the inner electrode-membrane interface, a water layer test was performed for both potassium and nitrate electrodes. To do this, the ISEs’ potential was first measured in 0.1 mol L^−1^ KNO_3_ (for K^+^-ISEs) and NaNO_3_ (for NO_3_^−^-ISEs) for a period of about 2 h, and then the electrodes were placed in a solution containing the interfering ions for a period of 3 h. This was 0.1 M NaNO_3_ for the potassium electrodes, while for the nitrate electrodes, it was 0.1 M CH_3_COONa. After this time, the electrodes were placed back in the main ion solution for another hour. In the case of the presence of an aqueous layer in the initial phase of the potential measurement (in stage III—re-measurement in the main ion’s solution), a significant potential drift would be generated—as we can see in Figure 6—for gold microelectrodes, we do not observe such a phenomenon. This is an excellent result because even without a hydrophobic intermediate layer (which would reduce the risk of formation of an aqueous layer), we have not formed a potential-distorting aqueous layer between the inner electrode and the membrane for electrodes sensitive to both potassium and nitrate ions. This can be explained by the fact that, when using GMA, the physicochemical conditions at the membrane-substrate electrode interface differ from those at macroelectrodes. Each of the microelectrodes in a GMA is micrometre-sized; consequently, the volume of any water layer that might form is extremely small, and it is therefore impossible for a ‘continuous water layer’ to form, which would cause potential drift. In this type of design, the membrane makes point contact with hundreds of small electrodes, so even if micro-points form in the water layer, they will not affect the electrodes in the same way as a conventional water layer. Furthermore, van der Waals forces come into play, which, in small structures, ensure good adhesion of the membrane to the substrate.

### 3.5. Reproducibility of SCISEs Based on GMA

To evaluate the reproducibility of GMA-based SCISE, four electrodes of each type were fabricated, and the potentiometric response was determined under the same conditions (mixed solution, calibration in the direction of increasing concentrations). Based on the results obtained, the variability of the calibration curve slope and the E^0^ potential were determined. The determined values of the E^0^ parameter were 312.3 ± 3.2 and 61.4 ± 5.1 mV for the GMA/ISM-K^+^ and GMA/ISM-NO_3_^−^, respectively, whilst the slope of the response curve was 56.21 ± 0.23 and 55.88 ± 0.11 mV/Dec for the GMA/ISM-K^+^ and GMA/ISM-NO_3_^−^, respectively. The low variability of these parameters indicates good reproducibility in the fabrication of electrodes using microelectrode array as a substrate.

### 3.6. Electrical Parameters of Electrodes Investigated by Chronopotentiometry

**Figure 7:**
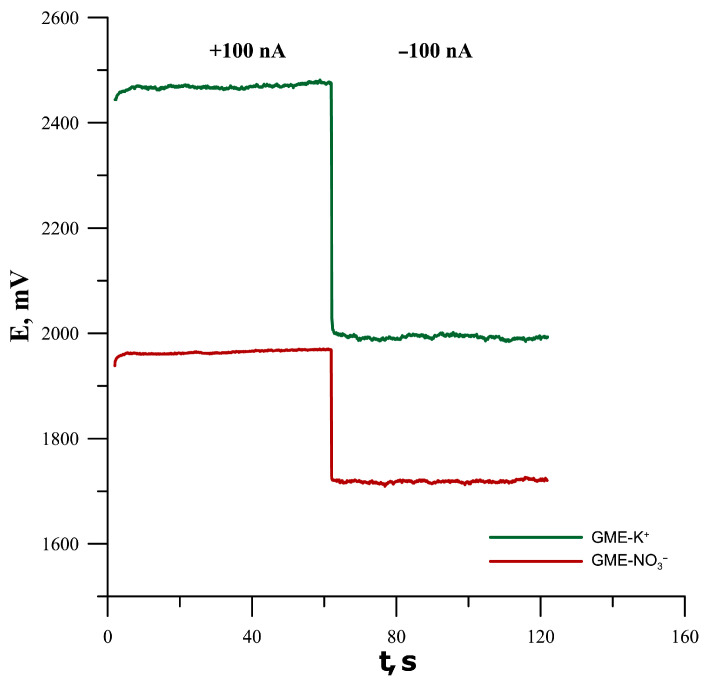
Chronopotentiograms of GMA/ISM-$K^{+}$ and GMA/ISM-${\mathrm{NO}}_{3}^{-}$ electrodes.

The constant-current chronopotentiometry method was used to determine the short-time potential stability under applied current, as well as the capacitance and total resistance of the tested electrodes based on the GMA substrate. Chronopotentiograms recorded in a KNO_3_ solution with a concentration of 10^−1^ mol L^−1^ for a current i = +100 nA for the first 60 s and for I = −100 nA for the next 60 s are presented in Figure 7. The potential jump obtained as a result of the change in applied current sign was used to estimate the total resistance of the electrodes (R), while the potential drift in time was used to determine the capacitance (C) according to the following formulas: R = E/i; C = i/(dE/dt), where E is the measured potential; i is the applied current; and t is time [11]. The determined electrical parameters of the studied electrodes are shown in Table 5, where it can be seen that both electrodes, despite containing no additional solid contact material, show good electrical performance. The values of these parameters are comparable to those of electrodes containing, for example, conductive polymers, carbon nanomaterials or other solid contact materials.

### 3.7. Analytical Application of Gold Microelectrode Array–Based ISEs

GMA/ISM-K^+^ and GMA/ISM-NO_3_^−^ electrodes were used for the analysis of tomato juice, for which the manufacturer declares 100% tomato content and no additives or preservatives. The analysis was performed on a sample without pretreatment, only after dilution (1:10) with ionic-strength buffer (0.05 mol L^−1^ sodium acetate). The content of both ions was determined using the standard addition method. The determined potassium concentration was 243.2 ± 7.9 mg/100 mL, and the nitrate content was 38.3 ± 1.6 mg/100 mL. The correct functioning of the electrodes was also confirmed by examining the recovery. For this purpose, the juice sample was fortified with KNO_3_ (50 mg/100 mL and 100 mg/100 mL), and the determination of potassium and nitrate was performed. The recovery values determined were in the range of 98.7–100.3%. In addition, the potassium content in the juice sample was determined using atomic absorption spectroscopy, yielding a result of 251.1 ± 13.5 mg/100 mL. The good agreement between the results obtained by the potentiometric method and the comparative method confirms the correct operation of the developed electrode and its analytical utility.

### 3.8. Performance Comparison of Gold Microelectrode Array–Based and Other Solid-Contact ISEs

A comprehensive review of the available research on various types of solid-contact ion-selective electrodes used for the determination of both potassium cations and nitrate anions has been made. The summary can be found in Table 6 and Table 7, where the properties of potassium and nitrate electrodes are compared with those of SC-ISEs using different substrates. Compared to other electrodes, we obtained slope values close to reference values. In terms of linearity and detection limit, our electrodes, despite the absence of an intermediate layer, achieved exceptionally good results. While our linear range did not always exceed that of other ISEs, this limitation is compensated for by the superior stability of our electrodes. A significant advantage of our GMA-based electrodes (for both K^+^-ISE and NO_3_^−^-ISE) is their exceptionally low potential drift, which was achieved without additional modifications, as is the case with the other electrodes shown in the tables. Our potential drift is among the best of the presented SC-ISEs, which is a significant step forward in ion-selective electrode technology.

## 4. Conclusions

The study presents a new type of internal electrode—gold microelectrode array (GMA). It has been successfully used to construct ion-selective electrodes sensitive to potassium and nitrate ions without employing an intermediate layer of solid contact. The use of a new type of substrate, namely a gold microelectrode array composed of several hundred (about 400) gold microelectrodes, provided an efficient charge transfer process between the membrane and the inner electrode. The resulting sensors exhibit remarkable repeatability and stability in readings, regardless of measurement method, so no control calibrations are required, significantly shortening and simplifying measurement procedures. A new internal electrode design and the absence of an intermediate layer ensure strong membrane adhesion to the substrate, extending the sensor’s lifespan. An additional advantage is that the amount of gold needed for the electrode substrate is negligible, making it cheaper to mass-produce than the glassy carbon disc electrodes used to date. The use of the electrodes for real-sample analysis and the achievement of recovery values close to 100% confirm their analytical suitability.

## Figures and Tables

**Table 1:** Basic analytical parameters of electrodes sensitive to potassium ions.

Electrode		**GMA/ISM-** $K^{+}$		**GCE/ISM-** $K^{+}$		**GE/ISM-** $K^{+}$	
Parameter	Calibration mode	non mix	mix	non mix	mix	non mix	mix
Slope [mV/Dec]	From 1 × 10^−6^ to 1 × 10^−1^	56.08	56.14	53.35	56.06	50.83	66.76
	From 1 × 10^−1^ to 1 × 10^−6^	56.55	56.62	51.15	53.16	55.19	65.29
Linearity range [mol L^−1^]	From 1 × 10^−6^ to 1 × 10^−1^	1 × 10^−5^–10^−1^	5 × 10^−6^–10^−1^	1 × 10^−5^–10^−1^	5 × 10^−6^–10^−1^	1 × 10^−5^–10^−1^	5 × 10^−6^–10^−1^
	From 1 × 10^−1^ to 1 × 10^−6^	1 × 10^−5^–10^−1^	5 × 10^−6^–10^−1^	1 × 10^−5^–10^−1^	5 × 10^−6^–10^−1^	1 × 10^−5^–10^−1^	5 × 10^−6^–10^−1^
Detection limit [mol L^−1^]	From 1 × 10^−6^ to 1 × 10^−1^	2.6 × 10^−6^	1.9 × 10^−6^	2.5 × 10^−6^	2.1 × 10^−6^	4.7 × 10^−6^	1.7 × 10^−6^
	From 1 × 10^−1^ to 1 × 10^−6^	2.4 × 10^−6^	1.6 × 10^−6^	2.4 × 10^−6^	2.0 × 10^−6^	3.6 × 10^−6^	1.9 × 10^−6^
E^0^ [mV]	From 1 × 10^−6^ to 1 × 10^−1^	310.36	311.01	358.41	361.31	451.83	557.75
	From 1 × 10^−1^ to 1 × 10^−6^	311.38	311.83	352.63	357.94	458.62	556.47

**Table 2:** Basic analytical parameters of electrodes sensitive to nitrate ions.

Electrode		**GMA/ISM-** ${\mathrm{NO}}_{3}^{-}$		**GCE/ISM-** ${\mathrm{NO}}_{3}^{-}$		**GE/ISM-** ${\mathrm{NO}}_{3}^{-}$	
Parameter	Calibration mode	non mix	mix	non mix	mix	non mix	mix
Slope [mV/Dec]	From 1 × 10^−6^ to 1 × 10^−1^	−55.48	−55.85	−55.44	−56.32	−59.29	−62.93
	From 1 × 10^−1^ to 1 × 10^−6^	−55.66	−56.14	−54.32	−57.00	−56.27	−57.27
Linearity range [mol L^−1^]	From 1 × 10^−6^ to 1 × 10^−1^	1 × 10^−5^–10^−1^	5 × 10^−6^–10^−1^	1 × 10^−5^–10^−1^	5 × 10^−6^–10^−1^	1 × 10^−5^–10^−1^	5 × 10^−6^–10^−1^
	From 1 × 10^−1^ to 1 × 10^−6^	1 × 10^−5^–10^−1^	5 × 10^−6^–10^−1^	1 × 10^−5^–10^−1^	5 × 10^−6^–10^−1^	1 × 10^−5^–10^−1^	5 × 10^−6^–10^−1^
Detection limit [mol L^−1^]	From 1 × 10^−6^ to 1 × 10^−1^	3.4 × 10^−6^	2.2 × 10^−6^	4.0 × 10^−6^	2.5 × 10^−6^	6.9 × 10^−6^	3.4 × 10^−6^
	From 1 × 10^−1^ to 1 × 10^−6^	2.5 × 10^−6^	1.0 × 10^−6^	4.0 × 10^−6^	1.9 × 10^−6^	3.2 × 10^−6^	2.4 × 10^−6^
E^0^ [mV]	From 1 × 10^−6^ to 1 × 10^−1^	61.8	60.1	110.13	119.21	72.94	97.60
	From 1 × 10^−1^ to 1 × 10^−6^	62.3	59.9	115.16	121.43	81.04	106.71

**Table 3:** The potential drift values of the tested electrodes determined during the first 10 min of measurement and during 3 h in 10^−3^ mol L^−1^ KNO_3_ solution.

Time Interval	Potential Drift (mV/min)					
	GMA/ISM-K^+^	GCE/ISM-K^+^	GE/ISM-K^+^	**GMA/ISM-** ${\mathrm{NO}}_{3}^{-}$	**GCE/ISM-** ${\mathrm{NO}}_{3}^{-}$	**GE/ISM-** ${\mathrm{NO}}_{3}^{-}$
0–10 min	0.0226	0.2975	0.3372	0.0151	0.0769	0.1863
10 min–3 h	0.0024	0.0372	0.1430	0.0083	0.1180	0.2441

**Table 4:** Comparison of the obtained selectivity coefficients for the tested electrodes.

Cation	${logK}_{K,M}^{pot}$		
	GMA/ISM-K^+^	GCE/ISM-K^+^	GE/ISM-K^+^
NH_4_^+^	−2.11	−2.11	−2.26
Li^+^	−5.12	−5.13	−5.07
Na^+^	−5.25	−5.20	−4.96
Mg^2+^	−5.41	−5.32	−5.15
Ca^2+^	−4.85	−4.83	−4.81
Anion	GMA/ISM-${\mathrm{NO}}_{3}^{-}$	GCE/ISM-${\mathrm{NO}}_{3}^{-}$	GE/ISM-${\mathrm{NO}}_{3}^{-}$
SO_4_^2−^	−3.25	−3.08	−2.97
CO_3_^2−^	−3.21	−3.13	−2.61
CH_3_COO^−^	−3.46	−3.28	−2.68
F^−^	−3.69	−3.14	−2.89
NO_2_^−^	−2.03	−1.92	−1.87
ClO_4_^−^	3.78	4.12	4.27

**Table 5:** Electrical parameters of the studied electrodes.

Electrode	Resistance (kΩ)	Potential Drift (µV/s)	Capacitance (µF)
GMA/ISM-K^+^	3872	175	571
GMA/ISM-${\mathrm{NO}}_{3}^{-}$	2397	193	518

**Table 6:** Summary of basic analytical parameters for tested electrodes and electrodes available in the scientific literature sensitive to potassium ions.

Ref	Electrode Material	Active Substance	Solid Contact	Slope (mV/dec)	Linear Range (mol L^−1^)	Detection Limit (mol L^−1^)	Potential Stability (μV/s)
This work	GMA	valinomycin	-	56.62	5.0 × 10^−6^–1.0 × 10^−1^	1.6 × 10^−6^	0.04 (i = 0) 175 (i = 100 nA)
[54]	GC	valinomycin	TTF-TCNQ	58.5	1.0 × 10^−6^–1.0 × 10^−1^	4.0 × 10^−7^	15.9 (i = 10 nA)
[55]	GC	valinomycin	CB-PtNPs	58.8	-	5.0 × 10^−7^	40.3 (i = 10 nA)
[56]	GC	valinomycin	OMC	55.1	6.5 × 10^−6^–6.2 × 10^−1^	5.4 × 10^−6^	3.33 (i = 1 nA)
[51]	GC	valinomycin	PEDOT-HQ	60.8	1.0 × 10^−6^–1.0 × 10^−1^	2.0 × 10^−7^	First 30 min—0.69, then 0.03 (i = 0)
[52]	GC	valinomycin	MoO_2_ microspheres	55.0	1.0 × 10^−5^–1.0 × 10^−3^	3.2 × 10^−6^	11.67 µV/s (i = 1 nA)
[57]	GC	valinomycin	MPCs	57.4	1.0 × 10^−5^–1.0 × 10^−1^	7.9 × 10^−7^	3.58 × 10^−3^ (i = 0) 31 (i = 1 nA)
[58]	GC	valinomycin	graphene	58.4	1.6 × 10^−6^–1.0 × 10^−1^	6.3 × 10^−7^	12.8 (i = 1 nA)
[59]	GC	valinomycin	CuO nanoparticles-MWCNTs	60.1	5.0 × 10^−7^–1.0 × 10^−1^	4.4 × 10^−7^	0.08 (i = 0)
[60]	GC	valinomycin	graphene	59.2	3.2 × 10^−5^–1.0 × 10^−3^	1.0 × 10^−5^	12 (i = 1 nA)
[30]	GC	valinomycin	perinone polymer	58.9	5.0 × 10^−6^–1.0 × 10^−1^	1.2 × 10^−6^	0.27 (i = 0)
[61]	GC	-	POT-CB	57.6	1.0 × 10^−6^–1.0 × 10^−1^	6.3 × 10^−7^	10.9 ± 0.5 (i = 1 nA)
[62]	GC	valinomycin	CNT-POT-hIrO_2_	57.3	1.0 × 10^−6^–1.0 × 10^−1^	-	0.012 (i = 0)
[63]	GC SPCE SPCE-MWCNT	valinomycin	PEDOT(CNT)	57.7 56.9 57.1	1.0 × 10^−6^–1.0 × 10^−1^	-	12 (i = 1 nA)
[64]	SPCE	valinomycin	PANI	60.5	1.0 × 10^−5^–1.0 × 10^−1^	1.6 × 10^−6^	0.78 (i = 0)
[65]	Graphite rods	valinomycin	FCB	59.9	1.0 × 10^−6^–1.0 × 10^−1^	1.0 × 10^−7^	0.12 (i = 0)
[66]	Pt	valinomycin	PPy/H-ZSM	54.2	1.0 × 10^−6^–1.0 × 10^−2^	7.1 × 10^−6^	0.036 (i = 0)
[67]	Cu wire	valinomycin	graphite-epoxy-hardener	44.0	5.0 × 10^−5^–1.0 × 10^−1^	4.0 × 10^−5^	-
[68]	Ni mesh	valinomycin	3DOM	59.2	1.0 × 10^−6^–1.0 × 10^−1^	1.6 × 10^−7^	0.003 (i = 0)

**Table 7:** Summary of basic analytical parameters for tested electrodes and electrodes available in the scientific literature sensitive to nitrate ions.

Ref	Electrode Material	Active Substance	Solid Contact	Slope (mV/dec)	Linear Range (mol L^−1^)	Detection Limit (mol L^−1^)	Potential Stability (μV/s)
This work	GMA	TDMAN	-	56.14	5.0 × 10^−6^–1.0 × 10^−1^	1.0 × 10^−6^	0.13 (i = 0) 195 (i = 100 nA)
[69]	GC	TDMAN	f-MWCNTs	−59.3	-	5.0 × 10^−7^	<0.014 (i = 0)
[70]	GC	TDMAN	CRGNO	−57.9	5.0 × 10^−5^–1.0 × 10^−1^	3.0 × 10^−5^	-
[71]	GC	TDMAN	PtNPs-CB	−58.6	1.0 × 10^−6^–1.0 × 10^−1^	5.0 × 10^−7^	0.6 (i = 1 nA)
[72]	GC	TDMAN	f-MWCNTs	−57.7	3.2 × 10^−6^–1.0 × 10^−1^	2.5 × 10^−6^	40 (i = 0)
[73]	GC	TDMAN	PANINFs-Cl	−56.8	1.0 × 10^−6^–1.0 × 10^−1^	3.2 × 10^−7^	1.47 × 10^−4^ (i = 0)
[74]	GC	TDMAN	MWCNTs-CuO nanoparticles	−60.4	1 × 10^−6^–1 × 10^−1^	5.1 × 10^−7^	0.085 (i = 0)
[75]	Ag/AgCl	THTDPCl	Ag/AgCl/Cl^−^	−60.1	1.0 × 10^−5^–1.0 × 10^−1^	2.8 × 10^−6^	0.013 (i = 0)
[76]	SPE	TDMAN	Co_3_O_4_ NPs	−56.8	1.0 × 10^−7^–1.0 × 10^−2^	1.0 × 10^−8^	1.6 (i = 1 nA)

GMA—gold microelectrodes array; GC—glassy carbon; TTF-TCNQ—tetrathiafulvalene-tetracyanoquinodimethane; CB-PtNPs—carbon-black-supporting platinum nanoparticles; OMC—ordered mesoporous carbon; POT-MWCNTs—nanocomposite of poly(3-octylthiophene-2,5-diyl) and multiwalled carbon nanotubes; PEDOT-HQ—conjugated redox polymer with hydroquinone (HQ) pendant groups covalently attached to the poly(3,4-ethylenedioxythiophene) (PEDOT) backbone; MPCs—monolayer-protected Au clusters; SPCE—screen-printed carbon electrode; POT-CB—nanocomposite of poly(3-octylthiophene-2,5-diyl) and carbon black; CNT-POT-_hIrO2carbon_ nanotubes-poly(3-octylthiophene-2,5-diyl)—hydrous iridium dioxide composite material; PEDOT(CNT)—poly(3,4-ethylenedioxythiophene) doped with carbon nanotubes; PANI—polyaniline; FCB—fullerene-enriched carbon black; dbdb-18-6—4′,4″(5″)-di-tert-butyldibenzo-18-crown-6-ether ionophore; PPy/H-ZSM—polypyrrole/zeolite composites; 3DOM—three-dimensionally ordered macroporous; TDMAN—tridodecylmethylammonium nitrate; SWCNTs—single-walled carbon nanotubes; MWCNTs—multiwalled carbon nanotubes; f-MWCNTs—lipophilic carbon nanotubes; CRGNO—chemically reduced graphene oxide; PtNPs-CB—carbon-black-supporting platinum nanoparticles; PANINFs-Cl—polyaniline doped with chloride ions; Nit^+^/NO_3_^−^—nitron–nitrate; GR-TTF(NO_3_)/NO_3_^−^—graphene/tetrathiafulvalene nanocomposite; ERGO/AuNPs nanocomposite—composite film of electrochemically reduced graphene oxide and gold nanoparticles; Ppy(NO_3_^−^)—polypyrolle doped with nitrate; Au NPs—gold nanoparticles; Co(Bphen)_2_(NO_3_)_2_—cobalt(II) complex with 4,7-diphenyl-1,10-phenanthroline; IL—ionic liquid; Ag/AgCl/Cl^−^—silver chloride electrode; THTDPCl—trihexyl- tetradecylphosphonium chloride; BEAMEX-S wire—standard-type irradiated polyethylene wire; TDAB—tetradodecylammonium bromide; CSPE—carbon screen-printed electrode; TDDA-NO_3_—tetradodecylammonium nitrate; GO—graphene oxide.

## Data Availability

The original contributions presented in this study are included in the article. Further inquiries can be directed to the corresponding author.
